# Computerised tomography-based planning with conventional total hip arthroplasty versus robotic-arm assisted total hip arthroplasty: study protocol for a prospective randomised controlled trial

**DOI:** 10.1186/s13063-020-04702-7

**Published:** 2020-09-10

**Authors:** Babar Kayani, Sujith Konan, Jenni Tahmassebi, Atif Ayuob, Fares S. Haddad

**Affiliations:** Department of Trauma and Orthopaedic Surgery, University College Hospital, 235 Euston Road, Fitzrovia, London, NW1 2BU UK

## Abstract

**Background:**

Robotic-arm assisted surgery aims to reduce manual errors and improve the accuracy of implant positioning during total hip arthroplasty. The objective of this study is to compare the accuracy of implant positioning, restoration of hip biomechanics, patient satisfaction, functional outcomes, implant survivorship, cost-effectiveness, and complications in conventional manual total hip arthroplasty (CO THA) versus robotic-arm assisted total hip arthroplasty (RO THA). Preoperative pelvic computerised tomography (CT) scans will be used to create patient-specific, virtual, three-dimensional reconstructions for surgical planning in both treatment groups.

**Methods and analysis:**

This prospective randomised controlled trial will include 60 patients with symptomatic hip osteoarthritis undergoing primary THA. Following informed consent, patients will be randomised to CO THA (control group) or RO THA (investigation group) at a ratio of 1:1 using an online random number generator. Observers will review patients at regular intervals for 2 years after surgery to record predefined study outcomes relating to the accuracy of implant positioning, hip biomechanics, postoperative rehabilitation, clinical progress, functional outcomes, cost-effectiveness, and complications. Primary and secondary objectives will be used to quantify and draw inferences on differences in the efficacy of treatment between the two groups. Intention-to-treat and per-protocol population analysis will be undertaken. Intention to treat relates to the allocated treatment (CO THA or RO THA), and per-protocol refers to the actual treatment received by the patient. The following statistical methods will be employed to analyse the data: descriptive statistics, independent *t* test, paired *t* test, analysis of variance, Fisher exact test, chi-square test, and graphical displays. Ethical approval was obtained from the London-Bromley Research Ethics Committee, UK. The study is sponsored by University College London, UK.

**Discussion:**

This study compares a comprehensive and robust range of clinical, functional, and radiological outcomes in CT-planned CO THA versus CT-planned RO THA. The findings of this study will enable an improved understanding of the differences in CO THA versus RO THA with respect to patient satisfaction, functional outcomes, implant survivorship, cost-effectiveness, and complications.

**Trial registration:**

ClinicalTrials.gov NCT04095845. Registered on 19 September 2019

## Background

Total hip arthroplasty (THA) is a highly successful surgical treatment for symptomatic hip osteoarthritis, which is performed in over 70,000 patients per year in the UK [1]. The procedure has well-established middle- to long-term clinical outcomes, and implant survivorship is greater than 90% at a minimum of 10 years’ follow-up [24]. Achieving accurate implant positioning and restoring native hip biomechanics are important technical objectives in THA. Studies have shown accuracy and reproducibility of achieving these surgeon-controlled factors during THA influences postoperative acetabular bone stock, abductor function, joint stability, soft tissue injury, impingement, bearing surface wear, and long-term implant survival [3, 5, 6, 12, 15, 18, 23]. Conventional manual THA (CO THA) uses radiographic templating, surgical alignment guides, and intraoperative landmarks such as the transverse acetabular ligament to help guide acetabular reaming and implant positioning. However, it is reported that only 38–47% of acetabular components are within the desired range of anteversion and inclination with CO THA, and low surgeon volume is a risk factor for inaccurate implant positioning [2, 3, 8, 10]. Suboptimal implant positioning outside of the acceptable safe ranges may lead to increased hip instability, poor restoration of native hip biomechanics, and premature component failure requiring more complex revision surgery [3, 10, 11, 15, 16].

Evolution in surgical technology has led to the development of robotic-arm assisted THA (RO THA), which aims to facilitate preoperative surgical planning, reduce intraoperative errors in bone resection, and improve the accuracy of implant positioning compared to CO THA. Preoperative computerised tomography (CT) scans of the pelvis and proximal femur are used to create virtual three-dimensional reconstructions of the patient’s anatomy. The surgeon uses this patient-specific computer-aided design (CAD) model to map optimal implant positioning to achieve the desired bone coverage, component positioning, hip biomechanics, and correction of any limb-length discrepancy. An intraoperative robotic device with audio, visual, and tactile feedback helps to execute the planned bone resection and implant positioning plan with a high level of accuracy. Existing reports comparing CO THA versus RO THA have shown conflicting outcomes [6, 14, 15, 20, 22]. Initial studies found RO THA was associated with improved functional outcomes as assessed using the Harris ship score, increased accuracy of acetabular implant positioning, better preservation of acetabular bone stock, improved restoration of native femoral offset, and reduced postoperative leg-length inequality compared to CO THA [6, 7, 9, 14, 15, 20]. However, further studies have shown no difference in CO THA versus RO THA with respect to functional outcomes, implant survivorship, and complications at short-term follow-up [4, 17, 19, 21]. Delays in the widespread implementation of RO THA have been attributed to the limited data showing any functional benefit with this procedure compared to an already established and highly cost-effective CO THA [7, 13].

Illgen et al. reviewed outcomes in 200 consecutive CO THAs followed by 100 consecutive RO THAs and found RO THA was associated with an additional 71% improvement in the accuracy of acetabular implant positioning compared with manual THA in the first year of use [9]. Acetabular implant positioning within Lewinnek’s safe zones (inclination, 30–50°; anteversion, 5–25°) was achieved in 30% of the first 100 consecutive CO THAs, 45% of the last 100 consecutive CO THAs, and 77% in the first 100 consecutive RO THAs (*p* < 0.001) [9]. Domb et al. conducted a retrospective radiological review of 50 patients undergoing CO THA versus 50 patients receiving RO THA [6]. All operative procedures were performed through the posterior approach. The study showed that all 50/50 (100%) RO THAs had acetabular cup positioning within Lewinnek’s safe zone compared to only 40/50 (80%) in the CO THA group (*p* = 0.001). Furthermore, 46/50 (92%) RO THAs had acetabular cup positioning within Callanan’s modified safe zone (inclination, 30–45°; anteversion, 5–25°) compared to only 31/50 (62%) in the CO THA group (*p* = 0.001). The odds ratio for an implanted acetabular cup out of Lewinnek’s safe zones was zero and Callanan et al. was 0.142 (95% CI 0.044–0.457). Nawabi et al. conducted a study on 12 cadaveric specimens that received CO THA on one side and RO THA on the contralateral side [20]. All procedures were performed using the posterior approach. The root-mean-square error for manual implantation was five times greater for acetabular cup inclination and 3.4 times greater for acetabular cup anteversion compared to robotic-arm assistance (*p* < 0.01). Tsai et al. conducted a retrospective study in which 12 patients undergoing RO THAs and 14 patients undergoing CO THAs had postoperative CT scans to assess implant positioning [25]. This study found that there was increased combined anteversion of 19.1 ± 11.7° in the RO THA group compared to 23.5 ± 23.6° in the CO THA group (*p* < 0.001). Cup inclination decreased by 16.5 ± 6.0° in the robotic THA group compared to 10.2 ± 6.8° in the CO THA group (*p* < 0.001).

The main limitations of existing studies comparing CO THA versus RO THA are that they are based on cadaveric specimens or retrospective clinical trials with limited data on functional outcomes, radiological results, or complications [6, 7, 9, 14, 15, 20]. Within each treatment group, different preoperative imaging modalities were used for surgical planning, operative procedures were performed with varying implant designs, and postoperative rehabilitation was not standardised. Observers recording outcomes were not blinded to the treatment group, and follow-up of outcomes and complications within each treatment group was limited to the early postoperative period [6, 7, 9, 14, 15, 20]. There is a need for high-quality evidence comparing CO THA versus RO THA. It is possible to improve on these previous studies by using the same preoperative planning technique in both treatment groups, prospectively randomising study patients to the treatment groups, and collecting data on a more comprehensive and robust range of clinical, functional, and radiological outcomes. All operative procedures will be undertaken using the standard posterior approach, sitting and standing spinopelvic radiographs will be used to assess patient-specific functional pelvic kinematics, and identical implant designs will be used in both treatment groups, which will help to blind observers recording radiological outcomes. The findings of this study will enable an improved understanding of differences in CO THA versus RO THA with respect to patient satisfaction, functional outcomes, implant survivorship, cost-effectiveness, and complications.

## Methods/design

### Objectives

The primary objective of this study is to compare the accuracy of achieving the planned centre of hip rotation in CO THA versus RO THA. This will be assessed within each treatment group by measuring the difference in the achieved centre of rotation on postoperative CT scanogram compared to the planned centre of rotation on preoperative pelvic CT scan. The study hypothesis is that RO THA will improve the accuracy of achieving the planned centre of rotation compared to CO THA.

The secondary objectives are to compare the following outcomes between the two treatment groups:

1. Accuracy of achieving planned component positioning
2. Accuracy of restoring planned hip biomechanics
3. Spinopelvic functional kinematics
4. Surgical efficiency
5. Postoperative functional rehabilitation
6. Functional outcomes
7. Quality of life
8. Range of motion
9. Resource use and cost-effectiveness
10. Complications

Specific study outcomes related to the primary and secondary objectives are discussed in the “Outcomes” section below.

### Trial design

**Fig. 1:**
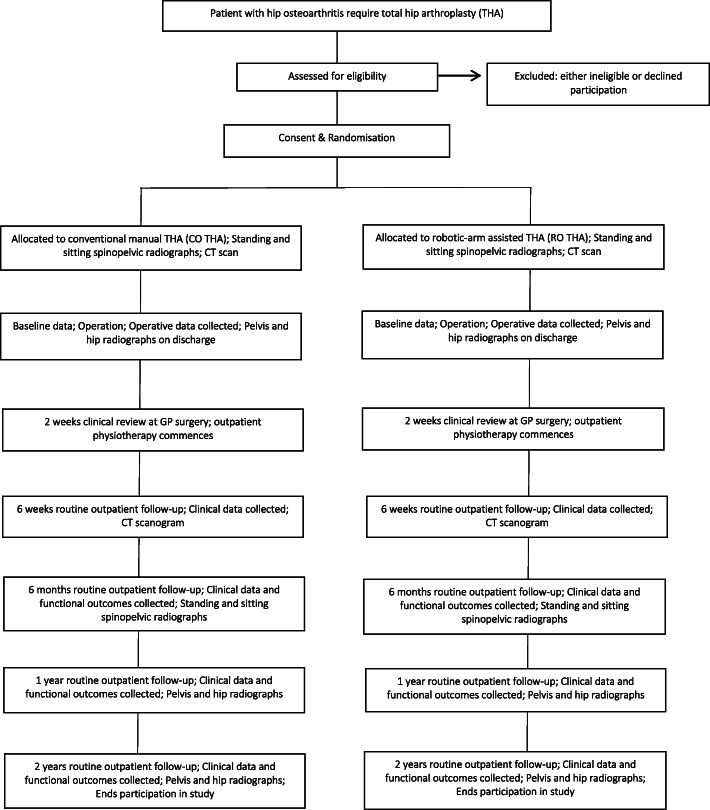
Patient enrolment flow chart

**Fig. 2:**
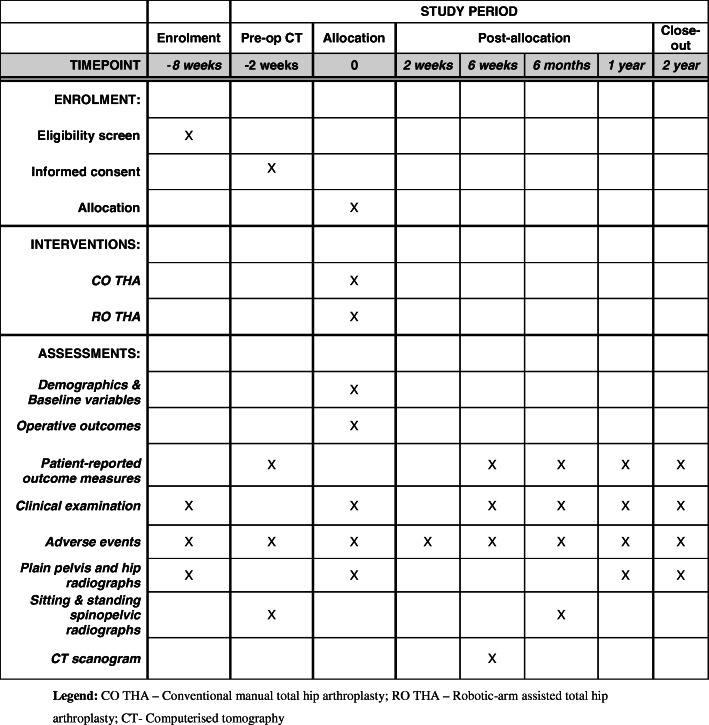
Schedule of enrolment, interventions, and assessments for all study patients

This study is a prospective, single-centre, randomised controlled trial. The study will be undertaken in the Department of Trauma and Orthopaedics, University College Hospital, 235 Euston Road, Bloomsbury, London NW1 2BU, UK. The study will include 60 patients randomly allocated to either CO THA (control group) or RO THA (investigation group). The study commenced patient recruitment in December 2018 and is expected to complete patient recruitment in December 2020. All patients will be followed up for 2 years after surgery, and therefore, the anticipated completion date for the study is December 2022. The study is sponsored by University College London, UK. The patient enrolment flow chart is presented in Fig. 1. The schedule of enrolment, interventions, and assessments for all study patients is shown in Fig. 2.

### Eligibility criteria

The inclusion criteria for this study are as follows: patient has symptomatic hip osteoarthritis requiring primary THA, patient fit for surgical intervention following review by surgeon and anaesthetist, patient aged between 18 and 80 years at time of surgery, patient able to give informed consent and agrees to comply with the postoperative review programme, and patient has sufficient mobility to attend follow-up clinics. The exclusion criteria for this study are as follows: patient undergoing revision surgery or second-stage THA, patients in whom the planned hip biomechanics are in a different position to the contralateral hip (e.g. developmental dysplasia of the hip or protrusio acetabuli), patient not suitable to have the planned study implants (e.g. requiring dual mobility component or cemented implants), patient had previous contralateral THA, patient is immobile or has another neurological condition affecting musculoskeletal function, patient already enrolled on another concurrent clinical trial, patient unable or unwilling to sign the informed consent form specific to this study, and patient unable to attend the study follow-up programme.

### Recruitment

Patients will be recruited from the orthopaedic outpatient clinic at University College Hospital, London, UK. All patients will be screened by the clinical team (orthopaedic consultant surgeon, clinical research fellow, and orthopaedic registrar) for study participation based on the predefined inclusion and exclusion criteria listed above. Patients that fulfil the eligibility criteria and express an interest to participate in the study will be provided with an ethics committee-approved patient information sheet. This provides details about the study treatment, follow-up, and contact details for further information. All members of the clinical team are familiar with the study and will address any preliminary questions about the study. Details of those patients expressing an interest to participate in the study will be recorded in the patient contact form and forwarded to the research physiotherapist. The research physiotherapist will phone the patient 4 weeks after this consultation to discuss any further questions and confirm if the patient would like to participate in the study.

### Consent

Informed consent will be obtained by the chief investigator or principal investigator when the patient attends for the preoperative planning CT scan. This is 6 weeks after the outpatient consultation for agreement to THA and 2 weeks before surgery. It is important to the data collection scheme that patients are able to follow commands, read, and interpret questions via questionnaires. For those who cannot hear, read, or understand English, an interpreter will be provided. The consent form describes the benefits and complications associated with THA and details additional risks associated with participation in this study, including additional radiation exposure (equivalent to two plain radiographs) compared to routine (non-research) patients undergoing THA. The consent form also explains that patients are free to withdraw from the study at any timepoint without their medical or legal rights being affected, their medical notes will be reviewed by the research team, and their general practitioners will be informed of their participation in the study.

### Allocation

After informed consent has been obtained, the research physiotherapist will randomise the patient into one of the two treatment groups using an online random number generator (www.random.org). A number from 1 to 60 can be randomly generated and will allocate a patient to one of the two arms of the study: 1–30 inclusive for the control group, 31–60 inclusive for the investigation group. The research physiotherapist will perform the randomisation procedure and store the designated treatment group for each patient on a password-encrypted file on the hospital computer. The operating surgeon will have this information communicated to him on the morning of surgery.

### Preoperative imaging

All patients will undergo preoperative imaging with sitting and standing spinopelvic radiographs, pelvic and hip radiographs, and CT scan of the pelvis and proximal femur. Preoperative templating will be undertaken by the operating surgeon. In both treatment groups, pelvic radiographs will be exported onto Traumacad software (Traumacad, Petach-Tikva, Israel) to template implant positioning and sizes for achieving the planned bone coverage, horizontal and vertical centres of rotation, acetabular and femoral offset, and leg-length correction. In all patients, the pelvic CT scan will be uploaded onto a computer software programme (Mako Surgical, Kalamazoo, MI, USA) to create a patient-specific CAD model of the patient’s osseous anatomy. The operating surgeon will use this virtual three-dimensional reconstruction to plan the operative procedure to restore the patient’s native hip biomechanics as guided by the contralateral side. In both treatment groups, the surgical objectives will be to restore the native horizontal and vertical centres of rotation, reproduce the native combined offset, restore natural femoral and acetabular version and inclination within Lewinnek’s and Callanan’s safe zones, and fully correct any pre-existing leg-length discrepancy. The computer robotic software will be used to calculate the required acetabular bone reaming, femoral osteotomy site, implant size, and implant positioning for achieving these surgical objectives.

### Surgical intervention

In patients undergoing CO THA, the femoral osteotomy site will be marked using the patient-specific CAD using measurements from the greater and lesser trochanters with the femoral neck cutting guide in place. An oscillating saw will be used to perform the osteotomy with Hohmann retractors protecting the surrounding soft tissues. The femoral osteotomy will be performed with the saw blade 45° to the femoral shaft and in the plane of the tibia. An entry point will be created in the proximal femur using a box chisel, with sequential reaming until contact with the cortical bone is felt, and then progressively larger raspers inserted into the proximal femur while maintaining the planned femoral version. Rasps will be used until the point where stability can be achieved with the definitive components. Sharp Hohmann retractors will be positioned over the anterior wall to lever the femur anteriorly and under the transverse acetabular ligament to expose the whole acetabulum for preparation. Soft tissues overhanging the acetabular circumference will be excised. Osteophytes will also be excised with an osteotome and the medial wall visualised. Sharp, hemispherical reamers will be used to remove the residual acetabular cartilage and expose the underlying subchondral bone. Reamers will be sequentially increased in size until the planned depth is reached. An external alignment guide will be attached to the cutting-edge reamer handle and acetabular impactor to improve the accuracy of acetabular cup positioning. The transverse acetabular ligament will be used as a fixed internal anatomical landmark for accurate positioning of the acetabular component within the safe zones of Lewinnek et al. and Callanan et al. to optimise stability and reduce the risk of dislocation. Any residual osteophytes will be removed at this stage using an osteotome and bone nibbler, and the trial prosthesis inserted to ensure satisfactory coverage and stability. Line-to-line technique for acetabular implantation will be used with implantation of the acetabular cup that is the same size as the last reamer used, and this will be augmented with two acetabular screws. The final femoral stem will then be implanted, femoral head applied to the taper, and hip reduced. The adjustment to a shorter or longer head can be performed at this stage.

In patients undergoing RO THA, three threaded registration pins will be inserted into the iliac crest for the attachment of the fixed pelvic array. One large screw will be inserted into the junction of the intertrochanteric ridge and lesser trochanter for the attachment of the femoral array, and a femoral checkpoint will be inserted just anterior to the greater trochanter. Femoral registration will be undertaken by registering and verifying the position of patient-specific anatomical landmarks displayed on the screen. Intraoperative measurements in the coronal plane as described by Murray will be displayed throughout the procedure. The femoral osteotomy will be marked using the probe and the osteotomy performed using an oscillating saw blade with Hohmann retractors protecting the surrounding soft tissues. The femur will be prepared with a box chisel, sequential reamers inserted, and progressively larger rasps inserted using the planned femoral version. The final rasp will be left in position and the femoral version recorded. The pelvic checkpoint will then be positioned outside the acetabular cavity in the bone just superior to the acetabular rim. Acetabular registration will be undertaken by registering and verifying the position of osseous landmarks displayed on the screen. The acetabular position and orientation may be fine-tuned based on intraoperative data on femoral version and inclination. The RIO robotic arm interactive orthopaedic system (Mako Surgical Corporation, Kalamazoo, USA) will be used to guide acetabular bone reaming within the confines of the haptic tunnel and implant the acetabular component into its final position and orientation as defined in the surgical plan. The final stem will then be implanted, trial head positioned onto the taper, and hip reduced. The femoral array will be inserted into the femoral screw, and the leg-length and offset change shown on the screen. The adjustment to a shorter or longer head can be performed at this stage.

All surgical procedures will be performed under the direct supervision of a single surgeon using the standard posterior approach. Trial implants will be used to assess hip stability, offset, soft tissue tension, and leg-length discrepancy prior to implantation of the permanent acetabular and femoral components in both treatment groups. Patients in both treatment groups will receive the standard or high-offset Accolade II femoral stem (Stryker Ltd., Mahwah, NJ, USA) and the trident acetabular shell (Stryker Ltd., Mahwah, NJ, USA). Patients in both treatment groups will undergo standardised inpatient and outpatient rehabilitation programmes. This will include six outpatient physiotherapy sessions at two-weekly time intervals. Patients will receive physiotherapy-supervised closed and open chain exercises to transition through four phases of rehabilitation with predefined milestones relating to the following: core strength and stability, lower limb proprioception, muscle strengthening, hip and knee range of motion, mobility with and without walking aids; ascending and descending stairs; and functional activities to return to pre-disease level of activity. Any deviations from the standardised proforma will be recorded and presented with the study findings.

### Outcomes

All study patients will undergo review by two observers (one orthopaedic registrar and one clinical research fellow) at 6 weeks, 6 months, 1 year, and 2 years following surgery. During these follow-up times, predefined clinical, functional, and radiological outcomes will be recorded by these observers using case report forms (CRFs). The following outcomes will be recorded in all study patients:

1. Accuracy of achieving the planned implant positioning and hip biomechanics (horizontal centre of rotation, vertical centre of rotation, acetabular offset, femoral offset, and leg-length discrepancy) as assessed using CT scanograms performed postoperatively at 6 weeks
2. Operating time (minutes)
3. Time to hospital discharge (hours)
4. Analgesia requirements during inpatient admission and postoperatively at 6 weeks, 6 months, 1 year, and 2 years
5. Patient-reported outcome measures including Oxford hip score (OHS), Harris hip score (HSS), Hip disability and osteoarthritis outcome score (HOOS), University College Hospital hip (UCH) score, Western Ontario and Mcmaster Universities Osteoarthritis Index (WOMAC), and University of California at Los Angeles hip (UCLA) score preoperatively and postoperatively at 6 weeks, 6 months, 1 year, and 2 years following surgery
6. Health-related quality of life as measured using European Quality of Life questionnaire with 5 dimensions for adults (EQ-5D) preoperatively and postoperatively at 6 weeks, 6 months, 1 year, and 2 years
7. Mobilisation distance (metres) and use of mobility aids during inpatient admission and postoperatively at 6 weeks, 6 months, 1 year, and 2 years
8. Range of movement (degrees) in the hip joint in the supine position as assessed with a goniometer during inpatient admission and postoperatively at 6 weeks, 6 months, 1 year, and 2 years
9. Resource use and cost-effectiveness including comparisons between the two treatment groups relating to operating time, theatre efficiency, equipment and sterilisation costs, analgesia requirements, inpatient rehabilitation, time to discharge, outpatient follow-up, additional imaging costs, and need for further surgery
10. Complications

### Blinding

It is not possible to blind study patients as RO THA is associated with an additional incision over the iliac crest for insertion of the acetabular registration pins. However, all observers recording radiological outcomes will remain blinded to the treatment group. Study patients will be identifiable with a unique study number. Only the research physiotherapist will have the key to identify individual patients and their respective treatment arm. Any documents related to the study will be archived directly at the study site by the research physiotherapist within a locked filing cabinet in a locked research office. This office has swipe card access with on-site security and 24-h CCTV surveillance. Patient data will be logged electronically using each patient’s unique identification number with computer software on an encrypted, password-protected research computer.

### Sample size

Prior to commencement of this study, a pilot study was performed to assess the accuracy of restoring the centre of rotation using CO THA versus RO THA [15]. Using data from this study, the mean centre of rotation was set at 3.7 mm for CO THA and 2.9 mm for RO THA, with standard deviation of 1.0 mm. Using a two-tailed, two sample *t* test with a power of 80% (1–*β*), significance level of 5%, and an effect size of 0.8, this study required 52 patients (26 in each treatment arm) to detect the minimum difference between the two treatment groups. To account for 10% attrition in the sample size during follow-up, the total sample size was set at 60 patients.

### Statistical analysis

The analysis of the per-protocol population will be considered the primary analysis. The differences between the CO THA and RO THA groups will be analysed by calculating the difference from baseline, per patient, and a two-sided confidence interval for the difference between the changes from baseline values will be calculated. This confidence interval will cover the true difference in the percentage change from baseline with a probability of 95%. The following statistical methods will be employed to analyse the data: descriptive statistics, independent *t* test, paired *t* test, analysis of variance, Fisher exact test, chi-square test, and graphical displays. Assumptions of normality will be tested with the D’Agostino test. Assumptions of homogeneity of variance will be tested with Levene’s test. If the distributional assumptions are (severely) violated, non-parametric techniques, such as Mann-Whitney’s test will be employed. In the event that RO THA is converted to CO THA intraoperatively, analysis will be performed using the intention-to-treat population and the treatment actually received by the patients. Intraoperative conversion from RO THA to CO THA will be documented and presented and published as part of the study. All statistical analysis will be undertaken by a blinded observer. Statistical significance is set at a *p* value < 0.05 for all analyses, and all statistical analysis will be performed using SPSS software version 25 (SPSS Inc., Chicago, IL, USA).

### Adverse events

Adverse events are defined as any untoward medical occurrence in a patient or study participant, which does not necessarily have a causal relationship with the procedure involved. A serious adverse event (SAE) is an adverse event that results in hospitalisation or prolongation of existing hospitalisation, persistent or significant disability or incapacity, life-threatening clinical sequelae, or death. All SAEs during the protocol treatment will be reported directly to the sponsor using the SAE web form. The chief investigator will also assess the SAE for severity, causality, seriousness, and expectedness using pre-existing criteria provided by the sponsor and inform the Data Safety Monitoring Board (DSMB) within 3 days of the initial observation of the event. The protocol treatment period is defined as the period from the day that the first study patient is recruited into the trial to the day that the final study patient has completed the 2-year follow-up. The chief investigator will also inform the London-Bromley Research Ethics Committee and local Health Research Authority within 3 days of the SAE taking place. Safety aspects of the study are closely monitored by the sponsor and DSMB using unblinded data for its judgement. In cases where the SAE arises due to a problem with the robotic device, Stryker Limited will also be notified within 2 days of the event taking place. The chief investigator will record the following: onset date, complete description of the event, severity, duration, action taken, and outcome for each SAE. The chief investigator will also provide regular updates of all SAEs to the London-Bromley Research Ethics Committee, local Health Research Authority, DSMB, and sponsor.

### Data management

On-site monitoring visits shall occur throughout the course of the clinical study by the chief investigator. The chief investigator shall permit and assist the sponsor (should they chose to monitor the study) to carry out verification of all study forms against data in the source documents, which shall occur as per the departmental policy for undertaking such activities. University College Hospital recognises that there is an obligation to archive study-related documents at the end of the study. The study master file will be archived at the University College London in accordance with the University College Hospital Standard Operating Procedure for Archiving of Investigator Site File (ISF) and Pharmacy Site File (PSF). It will be archived for a minimum of 5 years from the study end, and no longer than 30 years from the study end.

### End of protocol treatment

Reasons for going off study protocol include:

- Completion of last follow-up visit 2 years after surgery
- Patient non-compliance or withdrawal (the reason for discontinuation will be recorded in the case report form)
- Intercurrent death

All patients included into this study are free to withdraw from the study at any time without compromise to their future treatment. On withdrawal, patients will revert to the standard follow-up regimen for routine (non-study) THA at the study site. The end of study form will be completed and the reason for withdrawal documented. This form will also be completed if the patient is lost to follow-up or dies during the course of the study. Data to the point of discontinuation will be used for analysis.

### Monitoring

The chief investigator will monitor the progress of the clinical study in the form of monthly research meetings for those involved in the trial. The chief investigator will be responsible for day-to-day monitoring and management of the study. The UCLH/UCL/Joint Research Office, on behalf of UCL as sponsor, will monitor and conduct random audits on a selection of studies in its clinical research portfolio. Monitoring and auditing will be conducted in accordance with the Department of Health Research Governance Framework for Health & Social Care (April 2005) and in accordance with the sponsor’s monitoring and audit policies and procedures. As per the protocol, the principal investigator will email the sponsor twice yearly with the following: delegation log, adverse event log, deviation log, and any annual progress reports sent to the Research Ethics Committee. Any adverse events will also be included in any publications or presentations relating to the trial.

### Peer review

The study protocol has undergone independent external peer reviewer. The suggestions and recommendations for improvement to the study design were implemented. The reviewers and sponsor re-examined the revised protocol documents and confirmed that all queries and suggestions had been fully addressed.

## Discussion

Accurate restoration of hip biomechanics and implant positioning in THA are important surgeon-controlled variables that affect clinical outcomes and implant survivorship [3, 5, 6, 12, 15, 18, 23]. CO THA is performed using preoperative radiographic templates and intraoperative anatomical landmarks to manually guide implant positioning. However, this hand-held conventional technique is associated with 38–47% of patients receiving acetabular components outside of the planned safe ranges [2, 3, 8, 10]. The development of RO THA has enabled surgeons to use preoperative CT scans to create patient-specific surgical plans for achieving optimal implant positioning and hip biomechanics, and an intraoperative robotic-arm to execute this plan with a high level of accuracy [6, 14, 15, 20, 25]. This prospective randomised controlled trial compares a comprehensive and robust range of clinical, functional, and radiological outcomes between CO THA versus RO THA. All operative procedures will be undertaken using a standard posterior approach, sitting and standing spinopelvic radiographs will be used to assess patient-specific functional pelvic kinematics, and identical implant designs will be used in both treatment groups. The findings of this study will enable an improved understanding of differences in CO THA versus RO THA with respect to patient satisfaction, functional outcomes, implant survivorship, cost-effectiveness, and complications.

## Trial status

Protocol: version 3.0; date 26 October 2018

Patient recruitment date: 1 December 2018

Estimated completion of the recruitment date: 1 December 2020

Estimated completion of the final follow-up: 1 December 2022

## Registration

Registry name: ClinicalTrials.gov; reference: NCT04095845. Date registered: 19 September 2019.

URL: https://clinicaltrials.gov/ct2/show/NCT04095845?cond=mako&draw=2&rank=7.

## Data Availability

The datasets used and/or analysed during the current study are available from the corresponding author on reasonable request.

## Abbreviations

CO THA: Conventional manual total hip arthroplasty
DSMB: Data Safety Monitoring Board
EQ-5D: European Quality of Life questionnaire with 5 dimensions for adults
HHS: Harris hip score
HOOS: Hip disability and osteoarthritis outcome score
OHS: Oxford hip score
RO THA: Robotic-arm assisted total hip arthroplasty
SAE: Serious adverse event
SF-12: Short form health survey of 12 items
UCLA: University of California at Los Angeles hip score
WOMAC: Western Ontario and McMaster Universities Arthritis Index
